# Prediction of MoRFs based on sequence properties and convolutional neural networks

**DOI:** 10.1186/s13040-021-00275-6

**Published:** 2021-08-14

**Authors:** Hao He, Yatong Zhou, Yue Chi, Jingfei He

**Affiliations:** School of Electronic and Information Engineering, Hebei University of Technology, Tianjin, China

**Keywords:** Molecular recognition features, Intrinsically disordered proteins, Prediction, Convolutional neural network

## Abstract

**Background:**

Intrinsically disordered proteins possess flexible 3-D structures, which makes them play an important role in a variety of biological functions. Molecular recognition features (MoRFs) act as an important type of functional regions, which are located within longer intrinsically disordered regions and undergo disorder-to-order transitions upon binding their interaction partners.

**Results:**

We develop a method, MoRF_CNN_, to predict MoRFs based on sequence properties and convolutional neural networks (CNNs). The sequence properties contain structural and physicochemical properties which are used to describe the differences between MoRFs and non-MoRFs. Especially, to highlight the correlation between the target residue and adjacent residues, three windows are selected to preprocess the selected properties. After that, these calculated properties are combined into the feature matrix to predict MoRFs through the constructed CNN. Comparing with other existing methods, MoRF_CNN_ obtains better performance.

**Conclusions:**

MoRF_CNN_ is a new individual MoRFs prediction method which just uses protein sequence properties without evolutionary information. The simulation results show that MoRF_CNN_ is effective and competitive.

## Background

Recently, it has been recognized that many proteins, or regions of proteins, lack stable 3-D structures under apparently native conditions [1]. These proteins are called intrinsically disordered proteins (IDPs). Despite the lack of stable 3-D structures, IDPs have been confirmed to perform a variety of important biological functions, and thus are correlated with some diseases such as cancer and Alzheimer’s disease [2]. Molecular recognition features (MoRFs) act as an important type of functional region in IDPs. MoRFs permit interaction with structured partner proteins and can undergo disorder-to-order transitions upon interaction [3]. They generally vary in size and are up to 70 residues long, which are located within longer intrinsically disordered regions [4]. Usually, the unbound forms of MoRFs tend to adopt the conformation in the complex [5]. Because of the flexible structure, MoRFs can combine with their partner accurately. Therefore, they play important roles in regulatory processes and signal transduction [6].

MoRFs contain four subtypes: α-MoRFs, β-MoRFs, ɩ-MoRFs and complex-MoRFs [7]. When MoRFs bond, the four subtypes correspond to α-helices, β-strands, irregular secondary structures and multiple secondary structures respectively. The earliest prediction methods for MoRFs can only predict α-MoRFs, such as α-MoRF-PredI [8] and α-MoRF-PredII [9] based on neural network. Then, a number of methods have emerged to predict all kinds of MoRFs. MoRFpred [10] is the most used comparison prediction method. It contains five types of features which are gained from five disorder predictions [11–14], evolutionary profiles [15], selected amino acid indices [16], predicted B-factors [17] and RSA [18]. Then, a linear kernel support vector machine (SVM) is trained using these features to predict MoRFs. MoRF_CHiBi_ [17] is a representative method which does not rely on other predictors and evolutionary profiles, but obtains good prediction performance. It trains two SVM based on local physicochemical sequence properties, and combines the outcomes of them to predict MoRFs. MoRF_CHiBi_Light_ [19] utilizes Bayes rule to combine the scores obtained from ESpritz [20] and MoRF_CHiBi_. MoRF_CHiBi_Web_ [21] calculates the initial conservation score (ICS) by incorporating three values from the position specific scoring matrixes (PSSM). Then, the prediction results can be obtained by incorporating the ICS and the scores of ESpritz and MoRF_CHiBi_. OPAL [22] is also a combined prediction method. It first designs PROMIS [22] through training a SVM model based on half-sphere exposure, solvent accessible surface area and backbone angle information of MoRFs. Finally, OPAL is obtained by incorporating PROMIS and MoRF_CHiBi_. Besides, our previous work MoRF_MPM_ [23] and MoRF_MLP_ [24] also obtain good prediction results. MoRF_MPM_ selects 16 features and uses minimax probability machine to predict MoRFs. MoRF_MLP_ adds PSSM as evolutionary information to the 16 features selected by MoRF_MPM_, and trains MLPs separately for the two kinds of features. Then, their results are fused together to get the final result.

In this paper, we propose a new individual MoRFs prediction method, MoRF_CNN_, by training three convolutional neural networks (CNNs) based on three feature sets respectively, and then connecting them together. The first feature set obtains 16 sequence properties from our previous work MoRF_MPM_. The second and third feature sets, derived from MoRF_CHiBi_, contain 13 and 14 physicochemical sequence properties respectively. A preprocessing scheme is used to improve the effect of each feature set. Three windows of appropriate length are selected to calculate the features for each residue. Then, they are arranged into a feature matrix for conforming to the input form of CNN. The simulation results show that MoRF_CNN_ obtains better performance than other similar prediction methods.

## Results

### Datasets

In order to train our prediction method and compare with other methods, we utilize the widely used datasets that are created by Disfani et al. [10] They collect a lot of protein complexes concerning interaction between a protein and a small peptide from Protein Data Band [25] of March 2008. These complexes are filtered using a series of principles, and 840 protein sequences are selected. Then, they are divided into TRAINING and TEST sets which contain 421 and 419 protein sequences respectively. After that, using the same protocol, Disfani et al. create another test set TESTNEW which contains 45 protein sequences. To keep up with the comparison methods, we combine TEST and TESTNEW sets into TEST464. Besides, we also utilize TEST_EXP53 set [17] as another independent test set. TEST_EXP53 contains 53 protein sequences and is assembled by Malhis et al. The length of MoRFs in TRAINING and TEST464 sets is between 5 and 25 residues. However, TEST_EXP53 includes 729 MoRF residues from regions with up to 30 residues and 1703 from regions longer than 30 residues. Table 1 lists the specific information.

**Table 1:** Data sets used in this paper

Number	TRAINING	TEST	TESTNEW	TEST464	TEST_EXP53
Sequences	421	419	45	464	53
MoRFs Residues	5396	5153	626	5779	2432
non-MoRFs Residues	240,588	253,676	36,907	290,583	22,754
Total Residues	245,984	258,829	37,533	296,362	25,186

### Performance evaluation

We mainly utilize ROC (receiver operating characteristic) curve and AUC (the area under the ROC curve) to evaluate the performance. In addition, to evaluate the performance in detail, we also calculate the FPR (the false positive rate) at different TPR (the true positive rate). The FPR and TPR can be denoted as *FPR* = *TN*/*N*_*non*_, *TPR* = *TP*/*N*_*MoRF*_, where *N*_*non*_ and *N*_*MoRF*_ represent the total number of non-MoRFs and MoRFs residues, *TN* and *TP* represent the numbers of accurately predicted MoRFs and non-MoRFs residues, respectively.

### Impact of different windows

**Fig. 1:**
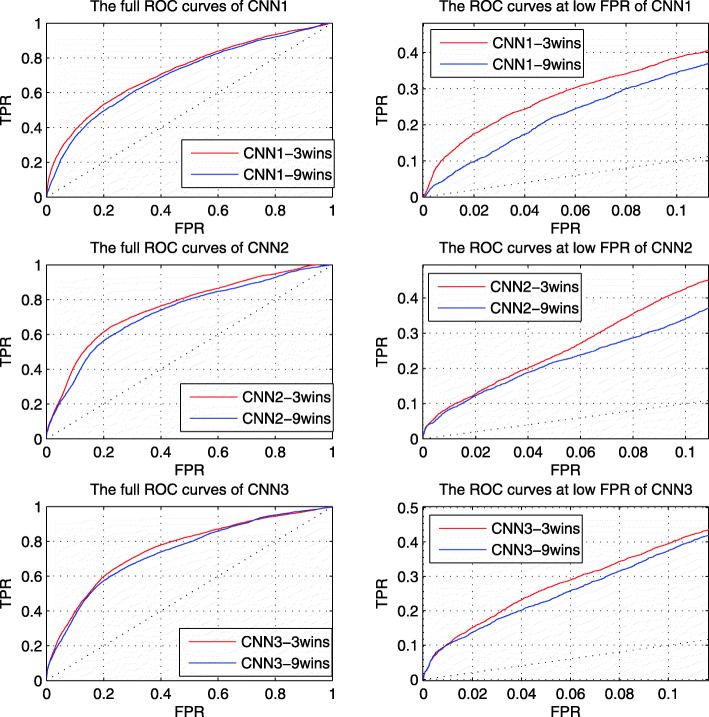
The ROC curves of each CNN with 3 windows and 9 windows. The blue curves are the results of 3 windows and the red curves are the results of 9 windows. The left figures are the full ROC curves. The right figures are the ROC curves at low FPR region

In the proposed method, we train three different CNNs based on three feature sets respectively. Based on our previous work, we select three windows for preprocessing with each feature set. The length 10 and 90 windows are used to highlight the characteristics of MoRFs and the surrounding environment, and the length 45 window is used to reduce the noise impact. In this section, we analyze the effect of increasing the number of windows on predictive performance. For comparison, we selected 9 windows in step 10 between windows of length 10 and 90. The performance of each CNN with 3 windows and 9 windows in TEST set is shown in Fig. 1. The left figures are the full ROC curves of them, and the right figures show their ROC curves at low FPR. Since the number of MoRF residue is much smaller than the number of non-MoRF residue, we will pay more attention to the prediction performance in the low FPR region.

From Fig. 1, the full ROC curves and the ROC curves at low FPR of CNN1, CNN2 and CNN3 of 3 windows are better than that of 9 windows. The results indicate that selecting too many windows will greatly increase the redundancy in the information, and thus increase the noise in the feature matrix. Therefore, only 3 windows with length of 10, 45 and 90 are selected for preprocessing and feature matrix calculation.

### Impact of different activation functions

**Fig. 2:**
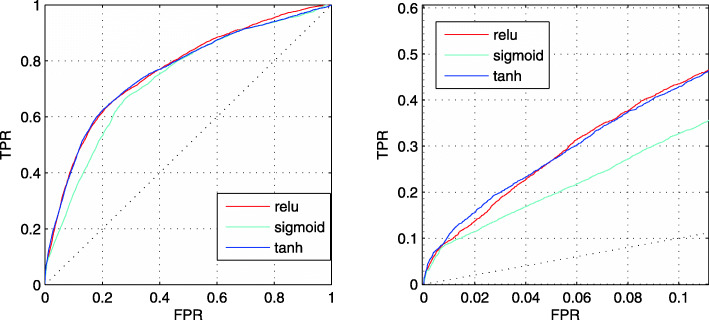
The ROC curves of CNN3 with different activation functions. The left figure is the full ROC curves. The right figure is the ROC curves at low FPR region

In this section, we compare the effects of different activation functions of each convolutional layer on the prediction performance. Figure 2 shows the prediction performance of ReLu function, sigmoid function and hyperbolic tangent function based on the third feature sets in TEST set.

From Fig. 2, the full ROC curve and the ROC curve at low FPR of ReLu function are similar to that of hyperbolic tangent function. However, the performance of sigmoid function is significantly worse. Thus, we select ReLu function as the activation function.

### Comparing CNNs and their combination

**Fig. 3:**
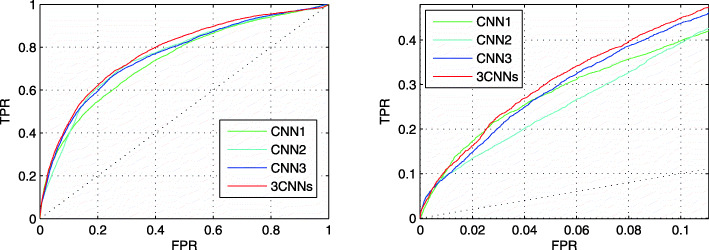
The ROC curves of three CNNs and their combined result. The red curves describe the combination result. The left figure shows the full ROC curves. The right figure shows the ROC curves at low FPR region

In this section, we compare the prediction performance of each CNN and the prediction performance of combining the prediction results of CNN directly. Figure 3 shows the prediction performance of them in TEST set. The left figure is the full ROC curves of them, and the right figure shows their ROC curves at low FPR. The red curves describe the average values of the prediction results of three CNNs. Through averaging, prediction performance improves a bit on both the full ROC curve and the ROC curve at low FPR.

### Impact of different convolutional layers

**Fig. 4:**
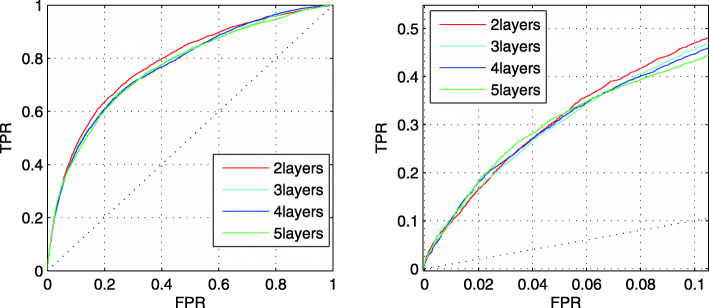
The ROC curves of the combined results with different convolutional layers. The left figure describes the full ROC curves. The right figure describes the ROC curves at low FPR region

We change the number of convolutional layers to analyze the influence on the prediction performance. Figure 4 shows the prediction performance of the combined results of three CNNs in TEST set with different convolutional layers.

From Fig. 4, the performance of 3 layers is similar to that of 2 layers. Besides, as the number of convolutional layers continues to increase, the prediction performance does not improve. Therefore, we still choose two convolutional layers for prediction.

### Comparing with other prediction methods

In this section, we compare our method, MoRF_CNN_, with MoRFpred, MoRF_CHiBi_, MoRF_CHiBi_Light_ and MoRF_MPM_. Among these methods, MoRFpred is a classical method, MoRF_CHiBi_ and MoRF_MPM_ are individual methods and do not use evolutionary information, MoRF_CHiBi_Light_ combines the scores of ESpritz and MoRF_CHiBi_. Because MoRF_CNN_ is a new individual MoRFs prediction method without evolutionary information, it is compared with similar types of methods. We use TEST464 and TEST_EXP53 sets for the performance comparison. Table 2 shows the AUC values of MoRF_CNN_ and other methods. From Table 2, MoRF_CNN_ gets higher AUC than MoRFpred, MoRF_CHiBi_, MoRF_CHiBi_Light_ and MoRF_MPM_ on both TEST464 and TEST_EXP53 sets. In addition, MoRF_CNN_ can process about 9000 residues per minute, which is similar to MoRF_CHiBi_Light_.

**Table 2:** AUC on TEST464 and TEST_EXP53

	MoRF_CNN_	MoRFpred	MoRF_CHiBi_	MoRF_CHiBi_Light_	MoRF_MPM_
TEST464	**0.787**	0.675	0.743	0.777	0.778
TEST_EXP53	**0.801**	0.620	0.712	0.799	0.758

We also compute the FPR values at different TPR to further analyze the performance of our method, as shown in Table 3. Obviously, MoRF_CNN_ obtains lower FPR values than MoRFpred as well as MoRF_CHiBi_, and obtains similar FPR values to MoRF_CHiBi_Light_ and MoRF_MPM_.

**Table 3:** FPR at different TPR on TEST464 and TEST_EXP53

	TPR = 0.2		TPR = 0.3		TPR = 0.4	
	TEST464	TEST_EXP53	TEST464	TEST_EXP533	TEST464	TEST_EXP533
MoRF_CNN_	**0.026**	**0.025**	**0.045**	**0.041**	**0.072**	**0.073**
MoRFpred	0.033	0.083	0.071	0.146	0.143	0.221
MoRF_CHiBi_	0.031	0.031	0.063	0.064	0.104	0.125
MoRF_CHiBi_Light_	0.020	0.016	0.040	0.043	0.073	0.068
MoRF_MPM_	0.027	0.025	0.047	0.056	0.074	0.096

## Discussion

The proposed method MoRF_CNN_ is an individual MoRFs prediction method which just uses protein sequence properties. These protein sequence properties are divided into three feature sets. The first feature set is from MoRF_MPM_ containing 13 physicochemical properties, 2 disorder propensities and topological entropy. The second and third feature sets, derived from MoRF_CHiBi_, contain 13 and 14 physicochemical properties respectively. To highlight the relationship between the residue and its surrounding environment, three windows are utilized to preprocess these three feature sets. Then, the preprocessed features are arranged into a feature matrix conforming to the input form of CNN. We train three CNNs based on three feature sets respectively, and then combine their results together. The simulation results show that MoRF_CNN_ is effective and competitive.

The following points enable MoRF_CNN_ to obtain good performance. First, the three feature sets of protein sequence properties are effective for predicting MoRFs. Second, the preprocessing process enhances the performance of these selected properties. Third, the constructed CNN prediction model can reflect the relationship between each feature and its neighboring features in the protein feature matrix, and find out more information from different features, and thus enrich the information proposed by protein sequences.

## Conclusions

In this paper, we propose a new individual MoRFs prediction method, MoRF_CNN_, based on sequence properties and convolutional neural networks. Comparing with other methods on TEST464 and TEST_EXP53 sets, MoRF_CNN_ obtains higher AUC than MoRFpred, MoRF_CHiBi,_ MoRF_CHiBi_Light_ and MoRF_MPM_. In addition, MoRF_CNN_ achieves lower FPR than MoRFpred and MoRF_CHiBi_, as well as similar FPR to MoRF_CHiBi_Light_ and MoRF_MPM_ when TPR is set to 0.2, 0.3 and 0.4. In the future, we will research different combination of the feature matrix and modify the topological structure of CNN to further improve the prediction performance.

## Methods

### Feature selection

We select three feature sets to describe the properties of MoRFs in this paper. The first feature set obtains 16 sequence properties which are from our previous work MoRF_MPM_. This feature set includes 13 physicochemical properties, 2 disorder propensities and topological entropy. Among them, the 13 physicochemical properties are selected from Amino Acid Index [16] using simulated annealing algorithm, the 2 disorder propensities are the Remark 465 and Deleage/Roux from GlobPlot NAR paper [26], the topological entropy is calculated after mapping the protein sequence to 0–1 sequence [27]. The second and third feature sets, derived from MoRF_CHiBi_, contain 13 and 14 physicochemical sequence properties from Amino Acid Index respectively.

In order to highlight the effect of these feature sets, we preprocess protein sequences according to each feature set. Taking the first feature set as an example, for a general protein sequence *w w* with length *L*, we select a window with the length of *N*(*N* < *L*) and fill *N*_0_ = ⌊(*N* − 1)/2⌋ zeros at the beginning and end of the sequence. Then, the sequence length becomes *L*_0_ = *L* + 2*N*_0_. We slide the window to intercept regions of length *N* with step of 1. For each intercept region, topological entropy is calculated through Eq. 14 of [27], and the remaining 15 sequence properties are calculated by the average value of mapped region of these properties. The calculated 16 dimensional vector **v**_*i*_(1 ≤ *i* ≤ *L*) is assigned to each residue in the region. After that, as the window slides, the vectors obtained by each residue are accumulated, and the average value is taken as the final feature vector for each residue under this window. This process can be represented as

1 $$ {\boldsymbol{x}}_j=\left\{\begin{array}{l}\frac{1}{j+{N}_0}\sum \limits_{i=1}^{j+{N}_0}{\mathbf{v}}_{\boldsymbol{i}},1\le j\le {N}_0\\ {}\frac{1}{N}\sum \limits_{i=j+{N}_0-N+1}^{j+{N}_0}{\mathbf{v}}_{\boldsymbol{i}},{N}_0<j\le L-{N}_0\\ {}\frac{1}{L_0-j-{N}_0+1}\sum \limits_{i=j+{N}_0-N+1}^{L_0-N+1}{\mathbf{v}}_{\boldsymbol{i}},L-{N}_0<j\le L\end{array}\right. $$

We can get a 16 dimensional feature vector for each residue under one window. In this paper, we choose several windows to preprocess. In order to conform to the input characteristics of CNN, we combine the feature vectors calculated from different windows into a feature matrix for each residue. Then, each residue can obtain a *N*_*win*_ × 16 feature matrix for the first feature set, where *N*_*win*_ denotes the number of windows. Similarly, each residue can obtain *N*_*win*_ × 13 and *N*_*win*_ × 14 feature matrices for the second and third feature sets.

Based on our previous work, we select three windows of length 10, 45, and 90 for preprocessing. Among them, the short window is used to highlight the characteristics of MoRFs, the long window is used to highlight the characteristics of MoRFs surrounding environment, and the middle window is used to reduce the noise impact brought by the long window.

### Prediction model

**Fig. 5:**
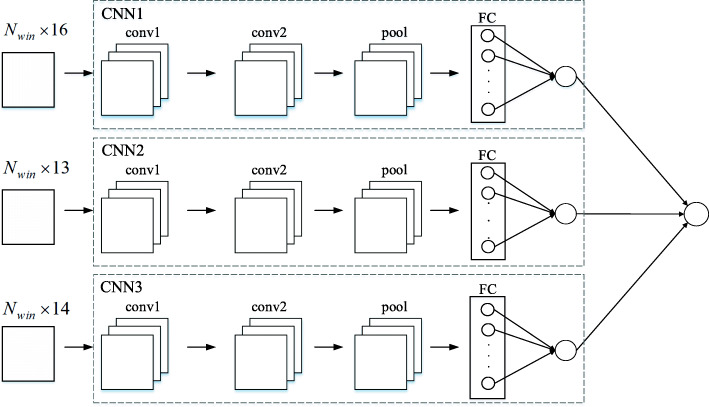
The structure of prediction model. Three CNNs are trained for three different feature sets. The finally prediction result is obtained by combining three CNNs results

We utilize the TRAINING set to train our prediction model. Three CNNs (CNN1, CNN2 and CNN3) are trained based on the selected three feature sets respectively. The finally prediction result is obtained by the average values of three CNNs results. Figure 5 shows the structure of prediction model.

**Fig. 6:**
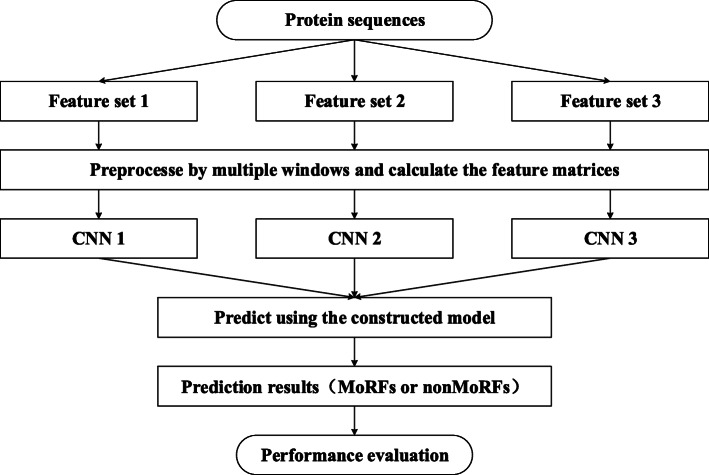
The detailed paradigm of the proposed method. Based on the selected three feature sets, the protein sequence is preprocessed to conform to the input characteristics of CNN. Then, it is predicted by the constructed CNNs

Each CNN contains two convolutional layers and one pooling layer as well as one fully connected layer. The activation function of each convolutional layer is ReLu function, and the activation function of the output layer is sigmoid function. In each convolution layer, the convolution step is 1 and performs same padding with zero. The parameters of conv1 and conv2 are set to 2 × 2 × 1 × 16 and 2 × 2 × 16 × 8 respectively. The pooling layer uses max pooling with 2 × 2 filter. In the designed CNN, the gradient descent algorithm is replaced by Adam algorithm [28] in the backward propagation to update parameters. In order to improve the operation speed, mini-batch is used to update parameters. That is, the sample set is divided into multiple subsets of equal scale for the each iteration, and each subset is used to calculate the gradient and update parameters one by one. In order to present our method more visually, combined with the feature selection, Fig. 6 shows the detailed paradigm of the proposed method.

## Data Availability

The datasets supporting the conclusions of this article are available on the references [10, 29].

## Abbreviations

MoRF: molecular recognition feature
CNN: convolutional neural network
IDP: intrinsically disordered protein
SVM: support vector machine
ICS: initial conservation score
PSSM: position specific scoring matrixes
ROC: receiver operating characteristic
AUC: the area under the ROC curve
FPR: the false positive rate
TPR: the true positive rate
